# Dietary patterns and metabolic dysfunction-associated fatty liver disease in China’s multi-ethnic regions

**DOI:** 10.1186/s41043-023-00485-0

**Published:** 2023-12-13

**Authors:** Xingren Zhu, Nima Qucuo, Ning Zhang, Dan Tang, Yifan Hu, Xiaofen Xie, Xing Zhao, Qiong Meng, Liling Chen, Xiaoman Jiang, Duoji Zhuoma, Qibing Zeng, Xiong Xiao

**Affiliations:** 1 Sichuan University, Chengdu, China; 2 Tibet Center for Disease Control and Prevention, Lhasa, China; 3 School of Public Health, Kunming Medical University, Kunming, China; 4 Chongqing Municipal Center for Disease Control and Prevention, Chongqing, China; 5 Chengdu Center for Disease Control and Prevention, Chengdu, China; 6 Tibet University, Lhasa, China; 7 School of Public Health, the key Laboratory of Environmental Pollution Monitoring and Disease Control, Ministry of Education, Guizhou Medical University, Guiyang, China

**Keywords:** MAFLD, Dietary pattern, Multi-ethnic, West China, Metabolic dysfunction

## Abstract

**Background:**

The prevalence of metabolic dysfunction-associated fatty liver disease (MAFLD) has been rising rapidly in western China. Diet acts as an effective therapy for MAFLD. However, there has been scarce research on the association between a posteriori diet patterns (DPs) and MAFLD in this region.

**Method:**

We identified three a posteriori DPs which were “Sichuan Basin pattern” characterized by a high intake of fish/seafood, poultry, fresh fruit and vegetables, indicating a balanced and modern DP; the “Yunnan–Guizhou Plateau dietary pattern” characterized mainly by a high intake of animal oil and salt, indicating an agricultural and poor DP; and the “Qinghai–Tibet Plateau dietary pattern” characterized by a high intake of coarse grains, wheat products, tubers and tea, respectively, indicating a high-altitude DP. Then, we performed marginal structural models that combined logistic regression and inverse probability exposure weighting (IPEW) to examine the associations between MAFLD and these a posteriori DPs.

**Result:**

We found the “Yunnan–Guizhou Plateau dietary pattern” revealed stronger positive association (OR = 1.50, 95% CI 1.40–1.60) with MAFLD than that of the “Qinghai–Tibet Plateau dietary pattern” (OR = 1.21, 95% CI 1.14–1.30). In contrast, the “Sichuan Basin dietary pattern” showed no significant association with MAFLD. In the further stratified analysis, we found those above associations were stronger in ethnic minorities and rural residents than their counterparts.

**Conclusion:**

Our study implied the unfavourable effects of “Yunnan–Guizhou Plateau dietary pattern” on MAFLD and provided evidence that reducing the intake of oil and sodium may be optimal for MAFLD control in the multi-ethnic region in western China.

**Supplementary Information:**

The online version contains supplementary material available at 10.1186/s41043-023-00485-0.

## Introduction

Metabolic dysfunction-associated fatty liver disease (MAFLD), formerly named non-alcoholic fatty liver disease (NAFLD), has emerged as the most common cause of chronic liver disease, affecting over one quarter of the population worldwide [1, 2]. In China, the prevalence of MAFLD is reported to be 29.2% [3], mainly due to sharp changes in lifestyle and diet [4]. In western China, where the majority of ethnic minorities live, the prevalence of MAFLD ranges from 21.8 to 26.1% [5, 6]. Of note, during the last decade, the prevalence of MAFLD in this region has doubled according to previous studies [6, 7].

Despite the alarming data, currently there is no pharmacotherapy available for MAFLD. Diet is the main driver of hepatic triglyceride accumulation [8] and thus may act as an effective therapy for MAFLD prevention and treatment [9, 10]. Considerable research has placed interest on the relationship between diet and MAFLD, but most of these studies have focused on either nutrients or some a priori dietary patterns [11, 12]. Single food or nutrient studies were not able to take complex combinations of foods into account [13]. While a priori dietary patterns, such as the Mediterranean diet (MED), Healthy Eating Index (HEI) and Dietary Approaches to Stop Hypertension (DASH) [14–17], have all been developed based on Western-like diets from developed countries, they may be not suitable for the multi-ethnic region in western China. A posteriori dietary patterns can represent the actual dietary habits of different regions, which could better provide insights into optimal dietary advices [18]. However, the effects of a posteriori dietary patterns on MAFLD in the multi-ethnic region in western China remains unclear.

To the best of our knowledge, there are no available evidence on the association between a posteriori dietary patterns and MAFLD in western China, although clear knowledge of this is critical for policy-making regarding MAFLD prevention [19]. The China Multi-Ethnic Cohort (CMEC) is a large-scale epidemiological study which has recruited nearly 0.1 million participants in western China with huge diversity in socioeconomic status, ethnicity, etc. [20]. In this context, the CMEC study provides a unique opportunity to shed light on the above research gap. Therefore, in this present work we aimed to examine the associations between a posteriori DPs and MAFLD in the multi-ethnic region in western China.

## Materials and methods

### Study population

The present work is based on the baseline data of the China Multi-Ethnic Cohort (CMEC) study, and detailed information about the CMEC has been described elsewhere [20]. Briefly, a total of 99,556 participants were recruited from May 2018 to September 2019 in the multi-ethnic region in western China. For every participant, data on demographics, behaviours and diet were recorded face-to-face by trained staff via an electronic questionnaire. Blood, urine and X-ray tests were also conducted. This study was conducted according to the guidelines laid down in the Declaration of Helsinki, and all procedures involving human subjects/patients were approved by the local ethics committee and the Sichuan University Medical Ethical Review Board [ID: K2016038] and all participants provided informed consent.

For the current analysis, we focused on participants between the ages of 30–79 years. We excluded participants whose total energy intake was extreme (N = 2433) (for males: < 800 or > 4200 kcal/day; for females: < 600 or > 3500 kcal/day), as well as those with an implausible body mass index (BMI)(N = 172) (< 14 or > 45 kg/m^2^). Participants with no available information on diet or outcome were also excluded (N = 4656). Furthermore, to capture a more reasonable causal effect and eliminate potential reverse causality, participants with self-reported chronic hepatitis/cirrhosis, coronary heart disease, stroke, hypertension, hyperlipidaemia, diabetes or cancer diagnosed by physicians were excluded as well. The final study sample consisted of 66,377 participants. Details are provided in Additional file 1: Figure 1.

### Assessment of dietary intake and dietary patterns

Dietary intake information was assessed via a validated quantitative food frequency questionnaire (FFQ) [21]. The FFQ mainly includes 13 food groups (include rice, wheat products, coarse grain, tubers, red and processed meats, poultry, fish/seafood, eggs, fresh vegetables, soybean products, preserved vegetables, fresh fruits and dairy products). Participants were interviewed to recall the quantity (average number of grams per meal) and frequency (how many times per day, week, year) of each food group during the last 12 months. Information about alcohol, tea and soft drink consumption was also collected. The validity and reproducibility were accessed by performing 24-h dietary recall (1113 participants) and repeated FFQ (7516 participants), respectively. We used intraclass correlation coefficients to access the reproducibility, which ranged from 0.15 for fresh vegetables to 0.67 for alcohol. We used de-attenuated Spearman rank correlation coefficients to assess the validity, which ranged from 0.10 for soybean products to 0.66 for rice. More details could be found in our previous work [21].

A posteriori dietary patterns were identified by performing principal component factor analysis (PCFA) via varimax rotation [22]. In consideration of eigenvalues, variance explained, scree plot and interpretability, three patterns were finally obtained. Food groups with absolute factor loadings ≥ 0.35 were considered to contribute to the corresponding factor. For each dietary pattern, factor scores were assigned to participants by summing the standardized intake of each food group weighed by factor loadings. A higher score means a higher adherence to the corresponding dietary pattern.

### Assessment of outcome

We used the criteria for MAFLD that were recently proposed by a panel of international experts from 22 countries [1]. The criteria were based on hepatic steatosis detected by either imaging, biomarkers or liver histology plus one of the following three conditions: overweight/obesity, type 2 diabetes mellitus or the presence of metabolic dysregulation. Overweight/obesity was defined as BMI ≥ 23 kg/m^2^ (criterion for Asians). Metabolic dysregulation was defined as the presence of at least two of the following conditions: (1) waist circumference ≥ 90/80 cm in men/women; (2) blood pressure ≥ 130/85 mmHg; (3) plasma triglycerides (TG) ≥ 1.7 mmol/L; (4) plasma high-density lipoprotein cholesterol (HDL-C) < 1.0/1.3 mmol/L for men/women; and (5) prediabetes (fasting plasma glucose within 5.6–6.9 mmol/L or haemoglobin A1c within 5.7–6.4%). Notably, in this analysis, we removed the insulinemia test and the high-sensitivity C-reactive protein (hs-CRP) test from the original criteria, because they were not collected in our baseline survey.

### Assessment of covariates

To obtain potential confounders, a directed acyclic graph was constructed according to the protocol of “Evidence Synthesis for Constructing Directed Acyclic Graphs” (ESC-DAGs) [23]. Guided by the DAG, we adjusted for the following covariates in our final models, including gender, age, ethnicity, urbanicity, marital status, education level, income, occupation, smoking status, alcohol use, physical activity (metabolic equivalent tasks), total energy intake (kcal/day), menopause status, family history of cardiovascular diseases (CVD) and insomnia status. See more details in Additional file 1: Figure 2.

### Statistical analysis

The baseline characteristics of participants were presented according to MAFLD status (with/without), as well as according to quintiles of each DP. (Quintile 1 represents lowest adherence, and quintile 5 represents highest adherence.) Continuous variables were presented as mean and standard deviation. Categorical variables were presented as number and percentages. Analysis of variance (ANOVA) was used to describe mean difference for continuous variables, and Chi-squared test was used to examine proportion difference for categorical variables.

To examine the associations of DPs and MAFLD, marginal structural models that combined logistic regression and inverse probability exposure weighting (IPEW) were used [24]. The entropy balancing weighting method was adopted due to the preferable performance of covariate balances [25] (see Additional file 1: Figure 3). The DP scores were modelled as categorical variables with five levels, with the lowest level (first quantile) as the reference group. Multiple imputation was conducted for missing food group values (with 5 imputations).

Furthermore, we conducted stratification analyses to detect potential effect modifiers among subgroups, including age, gender, urbanicity, ethnicity and income level. Sensitivity analyses were conducted to assess the robustness of our findings. First, we repeated our analyses with traditional logistic regression adjusting for the same covariates. Second, we redefined the outcome based on the NAFLD criteria. Third, participants with self-reported chronic hepatitis/cirrhosis, coronary heart disease, stroke, hypertension, hyperlipidaemia, diabetes or cancer diagnosed by physicians were included in the analysis. Fourth, we conducted an analysis based on complete cases instead of the imputation data.

All analyses were performed with R Project for Statistical Computing version 4.1.1.

## Results

### Characteristics of dietary patterns

Three a posteriori dietary patterns were identified using PCFA, and detailed information is presented in Table 1. Overall, these three DPs were highly geography-related. The first DP, named “Sichuan Basin dietary pattern”, was predominant among participants in the Sichuan Basin and was characterized by a high intake of fish/seafood, poultry, fresh fruits and vegetables, eggs, dairy products and vegetable oil with relative low intake of animal oil, salt indicating a more balanced DP. The second DP, named “Yunnan–Guizhou Plateau dietary pattern”, was predominant among participants in the Yunnan–Guizhou Plateau and was characterized mainly by a high intake of animal oil, rice, salt, preserved vegetables and alcohol, with low intake of dairy products, coarse grains, vegetable oil, fresh fruit and eggs. The third DP, named “Qinghai–Tibet Plateau dietary pattern”, was predominant among participants in the Qinghai–Tibet Plateau and was mainly representative of a high intake of coarse grains, wheat products, tubers, tea, potatoes and legumes with low intake of animal oil, salt, fresh vegetables and fish/sea food. In total, these three a posteriori DPs explained 28.5% of the variance in dietary intake.

**Table 1:** Rotated factor-loading matrix for the three dietary patterns

Food groups	Dietary pattern		
	Sichuan Basin	Yunnan–Guizhou Plateau	Qinghai–Tibet Plateau
Animal oil	− 0.24	0.61	− 0.12
Rice	0.07	0.53	0.00
Salt	− 0.03	0.35	− 0.13
Preserved vegetables	0.07	0.35	0.22
Alcohol	0.11	0.30	0.11
Legumes	0.23	0.26	0.23
Fresh vegetables	0.43	0.25	− 0.06
Fish/seafood	0.60	0.19	− 0.03
Red meat (includes processed)	0.24	0.18	0.22
Potatoes	0.13	0.17	0.54
Poultry	0.56	0.16	0.08
Tea	− 0.06	0.01	0.52
Wheat products	− 0.05	− 0.03	0.64
Fresh fruits	0.49	− 0.07	0.02
Eggs	0.52	− 0.07	0.08
Vegetable oil	0.36	− 0.10	− 0.04
Coarse grains	0.02	− 0.29	0.65
Dairy products	0.50	− 0.44	0.07
Variance explained (%)	10.9	9.0	8.6

### Characteristics of study population

Overall, there were 10,706 participants with MAFLD out of the 66,377 total participants, and the prevalence in our study population was 16.13%. The demographic, anthropometric characteristics of the participants with and without MAFLD are presented in Table 2. Generally, there were significant difference in gender, ethnicity, region, physical activity, obesity, alcohol intake, smoking status, education level and annual income between subjects with and without MAFLD. Subjects with MAFLD were more likely to be female and Han majority, majority of subjects live in rural area, and they tended to have lower physical activity levels and higher WC as well as BMI; furthermore, they also tend to have higher alcohol consumption.

**Table 2:** Comparison of the characteristics of participants with and without MAFLD

Variables	Without MAFLD *n* = 5 5613	With MAFLD *n* = 10 706	*P* value
Age (years)	49.45 (11.11)	49.59 (10.23)	0.215
*Gender (%)*			< 0.001
Male	19 728 (35.5)	5145 (48.1)	
Female	35 885 (64.5)	5561 (51.9)	
*Ethnicity (%)*			< 0.001
Han majority	31 836 (57.2)	6535 (61.0)	
Ethnic minority	23 777 (42.8)	4171 (39.0)	
*Region (%)*			< 0.001
Urban	18 288 (32.9)	4781 (44.7)	
Rural	37 325 (67.1)	5925 (55.3)	
Physical activity (METs-h/day)^a^	27.90 (18.44)	24.92 (17.36)	< 0.001
*Central obesity*^*b*^* (%)*			< 0.001
Yes	11,831 (21.3)	6969 (65.2)	
No	43,645 (78.7)	3721 (34.8)	
BMI (kg/m^2^)	23.17 (3.02)	27.13 (3.08)	< 0.001
*Alcohol intake*^*c*^* (%)*			< 0.001
Heavy	3357 (6.0)	806 (7.5)	
Light	52,256 (94.0)	9900 (92.5)	
*Education level (%)*			< 0.001
No formal school	14 120 (25.4)	2488 (23.2)	
Primary school	13 835 (24.9)	2641 (24.7)	
Middle and high school	21 061 (37.9)	4180 (39.0)	
College or university	6596 (11.9)	1397 (13.0)	
*Annual income (%)*			< 0.001
< ¥12,000	9230 (16.6)	1537 (14.4)	
¥12,000–19999	10 178 (18.3)	1839 (17.2)	
¥20,000–59999	20 606 (37.1)	3851 (36.0)	
¥60,000–99999	8061 (14.5)	1706 (15.9)	
¥100,000–199,999	5979 (10.8)	1386 (13.0)	
> ¥200,000	1486 (2.7)	377 (3.5)	
*Smoking status (%)*			< 0.001
Never	42 999 (77.3)	7666 (71.6)	
Previous	10 554 (19.0)	2471 (23.1)	
Current	2060 (3.7)	569 (5.3)	

*BMI* body mass index

^a^METs-h/day: hours of metabolic equivalent tasks per day

^b^Central obesity was defined as waist circumference ≥ 90 cm in men or ≥ 80 cm in women

^c^Heavy alcohol intake was defined as ≥ 140 g/week for men and ≥ 70 g/week for women

The characteristics of participants across quintiles of each DP score are shown in Table 3. Overall, participants in the highest quantile of the Sichuan Basin dietary pattern were more likely to be female, younger, Han majority, urban residents and non-smoker, they also tend to have higher education level and income level, and they were less likely to have central obesity. Contrast to participants in the bottom quantile, participants in the highest quantile of the Yunnan–Guizhou dietary pattern were more likely to be male, older, ethnic minority, rural residents, they tend to have lower education level and income level, most of them are married and have a habit of heavy drink, and they also are less likely to have central obesity and to be a smoker. Characteristics of participants in highest quantile of Qinghai–Tibet Plateau dietary pattern are similar as those in highest quantile of Yunnan–Kweichow Plateau pattern, they were more likely to be male, older, ethnic minority, rural residents, they tend to have lower education level and income level, and they also tend to have lower education level and income level.

**Table 3:** Characteristic of participants across quintiles of dietary pattern scores

	Sichuan Basin		*	Yunnan–Kweichow Plateau		*	Qinghai–Tibet Plateau		*
	Q1	Q5		Q1	Q5		Q1	Q5	
Age	51.67 (11.08)	47.69 (10.73)		47.55 (11.44)	51.40 (10.11)		49.54 (10.86)	50.13 (10.98)	
Male sex (%)	4470 (33.7)	5662 (42.6)		3279 (24.7)	7022 (52.9)		3316 (25.0)	6611 (49.8)	
Han majority (%)	3763 (28.3)	11,311 (85.2)		8627 (65.0)	7347 (55.3)		6362 (47.9)	6766 (51.0)	
Rural residents (%)	11,572 (87.2)	5295 (39.9)		6332 (47.7)	10,605 (79.9)		9221 (69.5)	9399 (70.8)	
Physical activity (METs-h/day)^a^	29.56 (20.03)	24.43 (15.74)		21.24 (14.84)	30.18 (19.01)		28.41 (18.43)	26.51 (18.55)	
With central obesity^b^ (%)	4198 (31.7)	3413 (25.8)		3905 (29.5)	3378 (25.5)		3331 (25.1)	4857 (36.7)	
*Education level*									
No formal school	6050 (45.6)	1131 (8.5)		3124 (23.5)	3583 (27.0)		3990 (30.1)	4154 (31.3)	
Primary school	4059 (30.6)	2301 (17.3)		2083 (15.7)	4619 (34.8)		3227 (24.3)	3661 (27.6)	
Middle and high school	2776 (20.9)	7010 (52.8)		5130 (38.6)	4582 (34.5)		4597 (34.6)	4296 (32.4)	
University	390 (2.9)	2834 (21.3)		2939 (22.1)	492 (3.7)		1462 (11.0)	1165 (8.8)	
*Annual income (%)*									
< ¥12,000	3722 (28.1)	1043 (7.9)		1391 (10.5)	2876 (21.7)		2860 (21.6)	2013 (15.2)	
¥12,000–19999	3253 (24.5)	1508 (11.4)		2039 (15.4)	2783 (21.0)		2573 (19.4)	2721 (20.5)	
¥20,000–59999	4718 (35.6)	4570 (34.5)		4585 (34.6)	5290 (39.9)		4566 (34.4)	5145 (38.8)	
¥60,000–99999	1018 (7.7)	2857 (21.6)		2447 (18.5)	1339 (10.1)		1732 (13.1)	1767 (13.3)	
¥100,000–199999	454 (3.4)	2475 (18.7)		2110 (15.9)	790 (6.0)		1281 (9.7)	1233 (9.3)	
> ¥200,000	99 (0.7)	801 (6.0)		683 (5.2)	179 (1.4)		246 (1.9)	383 (2.9)	
*Occupation (%)*									
Primary industry	7280 (54.9)	2014 (15.2)		2411 (18.2)	7471 (56.3)		5177 (39.0)	5016 (37.8)	
Secondary industry	659 (5.0)	1310 (9.9)		774 (5.8)	1145 (8.6)		1075 (8.1)	796 (6.0)	
Tertiary industry	3818 (28.8)	6407 (48.3)		6722 (50.7)	3099 (23.3)		5099 (38.4)	4662 (35.1)	
Unemployed	1508 (11.4)	3534 (26.6)		3359 (25.3)	1557 (11.7)		1912 (14.4)	2794 (21.1)	
*Smoking status (%)*									
Never	10,623 (80.0)	9638 (72.6)		11,617 (87.5)	8248 (62.1)		11,301 (85.1)	9018 (67.9)	
Previous	2268 (17.1)	2946 (22.2)		1292 (9.7)	4337 (32.7)		1651 (12.4)	3526 (26.6)	
Current	385 (2.9)	692 (5.2)		367 (2.8)	691 (5.2)		324 (2.4)	732 (5.5)	
Without family history of CVD^c^ (%)	10,486 (79.0)	7975 (60.1)		8576 (64.6)	9668 (72.8)		9395 (70.8)	9459 (71.2)	
*Menopause*^*d*^* (%)*									
Premenopause	3814 (28.7)	4667 (35.2)		5992 (45.1)	2906 (21.9)		5077 (38.2)	3506 (26.4)	
Perimenopause	602 (4.5)	537 (4.0)		647 (4.9)	465 (3.5)		734 (5.5)	432 (3.3)	
Post-menopause	4389 (33.1)	2410 (18.2)		3357 (25.3)	2883 (21.7)		4149 (31.3)	2726 (20.5)	
Without insomnia	7238 (54.7)	7917 (59.9)		7988 (60.4)	7641 (58.0)		7234 (54.7)	7938 (60.1)	
Energy intake (Kcal/day)	10,775.52 (3,871.93)	15,921.89 (4,445.82)		11,860.37 (4,085.58)	16,475.08 (4,451.20)		10,953.17 (3,762.20)	16,410.08 (4,613.38)	
Heavy alcohol intake ^e^ (%)	489 (3.7)	1250 (9.4)		98 (0.7)	2397 (18.1)		248 (1.9)	1333 (10.0)	
Married or cohabit (%)	11,676 (87.9)	11,962 (90.1)		11,755 (88.5)	11,998 (90.4)		11,672 (87.9)	12,091 (91.1)	

*CVD* cardiovascular disease

Q: represent the quintile of dietary pattern score

*All *p* values are < 0.05

^a^METs-h/day: hours of metabolic equivalent tasks per day

### Dietary pattern and MAFLD

**Fig. 1:**
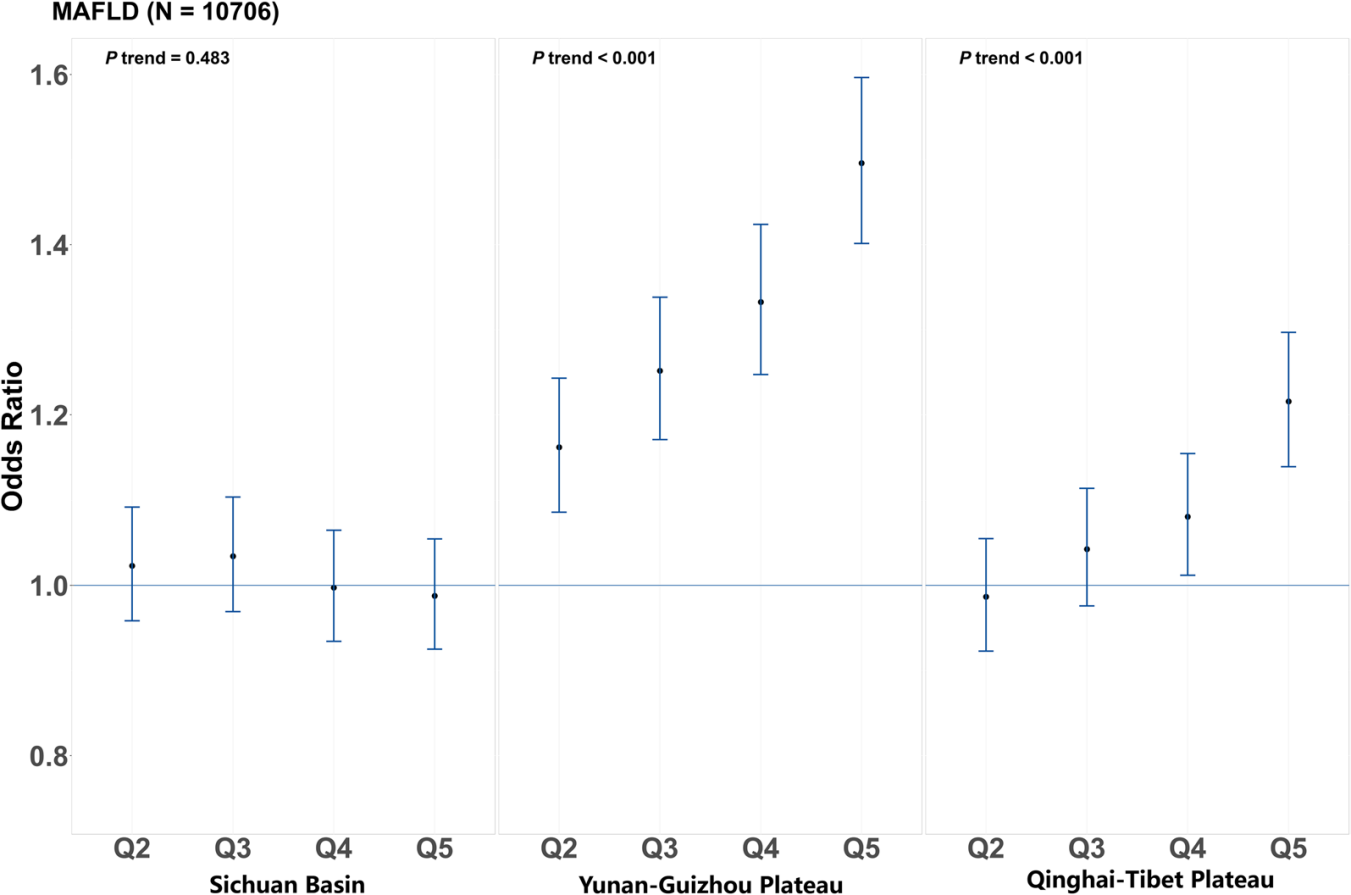
Estimated association between dietary patterns and MAFLD. Estimated association between dietary patterns and MAFLD according to quintiles of dietary pattern scores, with the lowest quintile as reference group. *N* in the brackets represent the number of MAFLD. Q2–Q5 represent the second to fifth quintiles of dietary pattern scores. The filled dots represent adjusted odds ratios, and the vertical blue lines represent 95% CIs

Figure 1 presents the associations between a posteriori DPs and MAFLD after adjusting for potential confounders. Overall, we found that the association between these three a posteriori DPs and MAFLD varied. More specifically, the Yunnan–Guizhou Plateau dietary pattern was positively associated with MAFLD, participants in the highest quantile of this pattern had a 50% greater risk of developing MAFLD (OR = 1.50, 95% CI 1.40–1.60, *p* for trend < 0.001) than those in the bottom category. The Qinghai–Tibet Plateau dietary pattern is also positively associated with MAFLD (OR = 1.21, 95% CI 1.14–1.30, *p* for trend < 0.0001), participants in the highest quantile had a 21% greater risk of developing MAFLD. However, the Sichuan Basin dietary pattern did not show significant association with MAFLD in this study.

**Fig. 2:**
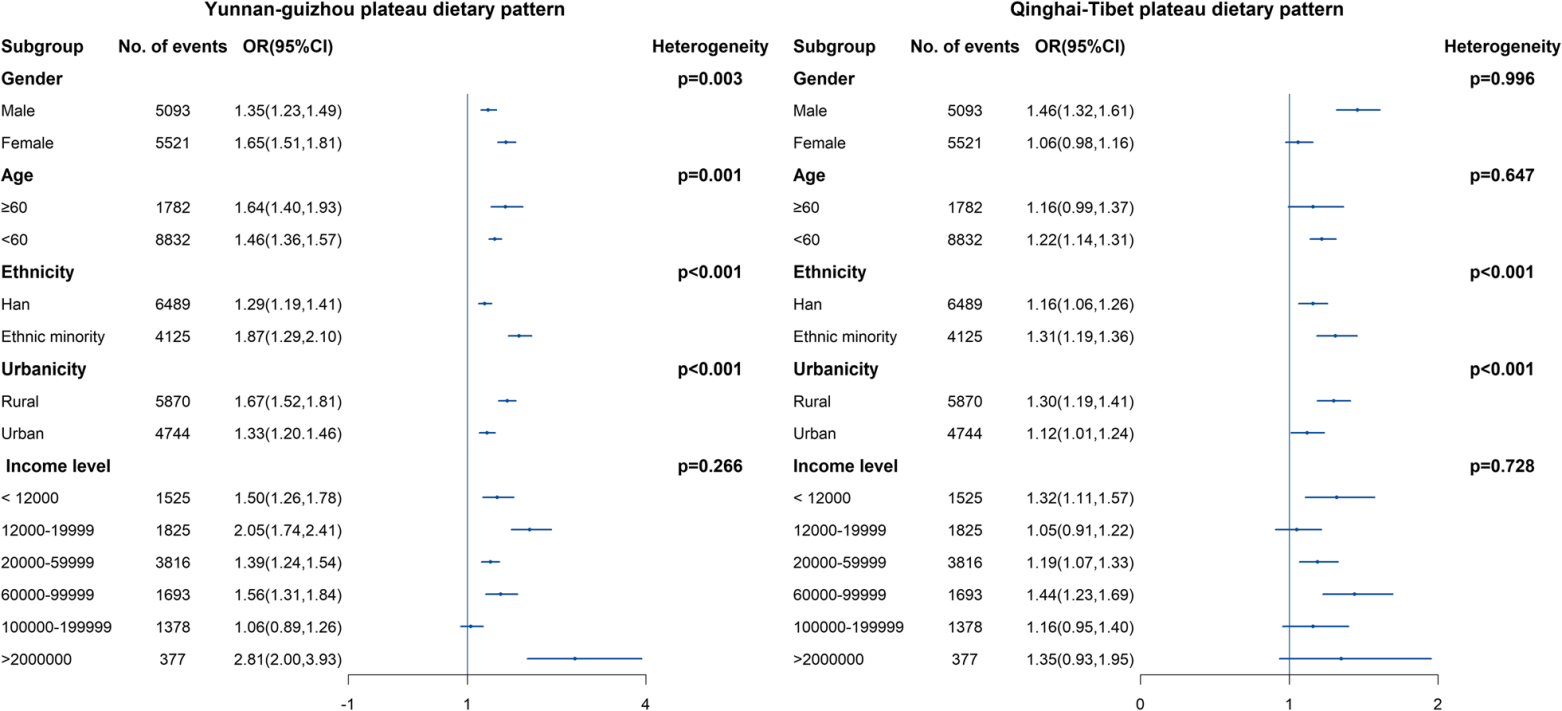
Stratified analysis of estimated association between dietary patterns and MAFLD. Stratified analysis of estimated associations between the Sichuan Basin dietary pattern or the Yunnan–Guizhou Plateau dietary pattern and MAFLD according to gender, age, ethnicity, region and household income, by comparing the highest with the lowest quintiles. Chi-squared tests were performed to examine heterogeneity among different subgroups. The filled blue dots represent adjusted odds ratios, and the vertical blue lines represent 95% CIs

Figure 2 shows the results of the stratification analyses result for the Yunnan–Guizhou Plateau dietary pattern and the Qinghai–Tibet Plateau dietary pattern. The result of Sichuan Basin Dietary Pattern was not presented due to it is not significant associated with MAFLD. For both a posterior DPs, we observed a stronger positive association. For Yunnan–Guizhou Plateau pattern, we found the association in rural residents (OR = 1.67, CI 1.52–1.81) is greater than in urban residents (OR = 1.33, CI 1.20–1.46), and the association in ethnicity minority (OR = 1.87, CI 1.29–2.10) is also greater than in Han majority (OR = 1.29, CI 1.19–1.41). Similarly, for Qinghai–Tibet Plateau pattern, we found the association in rural residents (OR = 1.30, CI 1.19–1.41) is greater than in urban residents (OR = 1.12, CI 1.01–1.24), and the association in ethnicity minority (OR = 1.31, CI 1.19–1.36) is also greater than in Han majority (OR = 1.16, CI 1.06–1.26). Both P values were less than 0.05. We did not observe this difference in gender subgroup.

The results of the sensitivity analyses are provided in Additional file 1: Figures 4–7, which were roughly the same as the main analyses, indicating that our findings were robust.

## Discussion

In this study, we found that the Yunnan–Guizhou Plateau dietary pattern and the Qinghai–Tibet Plateau dietary pattern were positively associated with MAFLD, while the Sichuan Basin dietary pattern was not significantly associated with MAFLD. And we also found that this positive association was greater in rural residents and ethnicity minority.

In the present study, we found that the prevalence of MAFLD was 16.13%, which was relatively low. In a previous study conducted in east China, the prevalence was 26.1% [26], and another meta-analysis [27] showed a prevalence of 29.1% in China. The reason why the prevalence was low in this population may as follows. Many risk factors have been identified in previous study [28, 29], which including age, obesity, genetic background, socioeconomic status, complications and lifestyle such as diet and physical activity. It has been well recognized that obesity is a vital contributor to MAFLD, several researches have studied the relationship between obesity and MAFLD [30, 31], and most studies concluded that obesity was positively associated with MAFLD. For our study population, the mean BMI was 23.81 and the ratio of have central obesity was 28.4% indicating a relative thin population which may contribute to the low prevalence of MAFLD. Genetic background is another well-studied risk factor for MAFLD [32–34], and it has been showed that the Uygur and Hui ethnic groups have higher rates of NAFLD than the other groups [27, 32]. Our study population consist 9 different ethnicity groups and there may be huge diversity in every aspect of these ethnicity such as genetic background, socioeconomic level, culture including lifestyle, dietary patterns, which could result in dramatic differences in the prevalence of NAFLD.

Our results showed the unfavourable role of the Yunnan–Guizhou Plateau dietary pattern which is characterized by high intake of animal oil, rice and salt. Our previous work has also revealed a strong positive association between this dietary pattern and MetS, a risk factor for MAFLD [21]. Our results were consistent with a body of published works. Animal oil is rich in saturated fatty acid (SFA), and it has proved that SFA was positively associated with MAFLD [35, 36]. In a study conducted in Chinese population, researchers found that high intake of SUA was positively associated with MAFLD (OR = 7.56. CI 3.55–15.88) [37]. Another study has also come into same conclusion by replacing SFA with unsaturated fatty acid (USFA) [38]. In a study enrolled in 28,438 participants from Korea, researchers found that high adherence to carbohydrate/rice-rich diet had a 1.63 to 1.88 times stronger association with MAFLD [39]. Another study that focuses on middle-aged Japanese has also found that high intake of rice was positively associated with MAFLD [40]. The undesirable role of salt for MAFLD has been recognized well [41]. A previous study based on Korea National Health and Nutrition Examination Survey concluded that high salt intake was related to high prevalence of MAFLD (OR = 1.49, CI 1.28–1.73). Similarly, a recent study involved in 23,867 Chinese participants has also come into the conclusion that salt intake was associated with increased NAFLD (OR = 1.60, CI 1.46–1.75) [42]. As for the Qinghai–Tibet Plateau dietary pattern, it is a bit more complex, the negative role of this dietary pattern was only observed in the highest two quintiles. This may due to the interaction between its mixed components. This dietary pattern was dominated by high intake of coarse grains, wheat products, tea and potatoes, and the characteristic of this pattern was that it was rich in both high carbohydrates and dietary fibre. According to previous studies, high intake of dietary fibre and tea may be benefit for MAFLD prevention [43–45], while the intake of high carbohydrates may be related to higher risk of MAFLD [39].

In our study, we also observed a stronger positive association between both Yunnan–Guizhou Plateau dietary pattern and Qinghai–Tibet Plateau dietary pattern with MAFLD in rural and ethnic minority populations. A few of studies have examined the heterogeneity of the association between dietary patterns and MAFLD [46], and they did not find heterogeneity across subgroups. Researches have confirmed that higher-quality food could reduce the risk of MAFLD [47, 48]. For the rural residents and ethnic minorities, most of them lived in plateau area with relatively low-level income, which can substantially restrict the affordability and availability of high-quality food [49]. In this distinct living environment, some featured foods were consumed much more such as potato, rice, as well as red meat and animal oil. Previous researches have indicated that high intake of foods list above was positively associated with MAFLD [50–52]. It has been proved that the dietary culture may differ in ethnicity and socioeconomic status [53], and this might also give rising to this heterogeneity of effect. In the rural and ethnic minority region, the cooking style, way of storage and the taste of food may differ from those of their counterparts. These might also explain the heterogeneity in associations to some extent.

This was the first and largest study to examine the associations between a posteriori DPs and MAFLD in the multi-ethnic region in western China. Our study sample consisted of multi-ethnic participants, which provided a unique opportunity to exclusively detect the relationship between dietary patterns and MAFLD in China’s multi-ethnic region. Besides, we performed the statistical analyses under the guide of the framework of causal inference, which could transparent confounder selection and effect estimation. However, there are limitations in our work. First, it was a cross-sectional study, so the association may not be causal. Second, in the assessment of MAFLD, objective indicators, including insulin and hs-CRP, were not collected for the sake of data availability, which may undermine the number of MAFLD patients. Third, the FFQ used in our study only included 13 crude food groups for feasibility. In this study, many participants were illiterate; often, they spoke different local languages and consumed distinct foods, so it is not be feasible to conduct a more detailed FFQ. Fourth, although we identified potential confounders using a DAG, residual confounders may remain.

## Conclusion

In conclusion, in this study we found that both the Yunnan–Guizhou Plateau dietary pattern which is characterized by a higher intake of animal oil, rice and salt and the Qinghai–Tibet Plateau dietary pattern which is characterized by high intake of coarse grain, wheat products, potatoes and tea were positively associated with MAFLD. This may suggest the unpreferable role of salt, animal oil and high carbohydrates in the progress of MAFLD, thereby providing insights into the future dietary intervention in the multi-ethnic regions in western China.

### Supplementary Information

**Additional file 1**. Additional Material.

## Data Availability

Data described in the manuscript, codebook and analytic code will be made available from the corresponding author (Xiong Xiao, Ph.D., email: xiaoxiong.scu@scu.edu.cn) upon reasonable request, with approval by the principal investigators of the China Multi-Ethnic Cohort study.

## Abbreviations

MAFLD: Metabolic dysfunction-associated fatty liver disease
SES: Socioeconomic status
DASH: Dietary Approaches to Stop Hypertension
BMI: Body mass index
WC: Waist circumference
CMEC: The China Multi-Ethnic Cohort Study
MED: Mediterranean diet
HEI: Healthy Eating Index
NAFLD: Non-alcoholic fatty liver disease
FFQ: Food frequency questionnaire
DAG: Directed acyclic graph
OR: Odds ratio
CI: Confidence interval
