# Supplementary material for: Dietary patterns and metabolic dysfunction-associated fatty liver disease in China’s multi-ethnic regions
Source: J Health Popul Nutr. 2023 Dec 13;42:141. doi: 10.1186/s41043-023-00485-0 (PMC10717100; doi:10.1186/s41043-023-00485-0)
Supplement: Supplementary file 1 — Additional file 1. Additional Material. [file 41043_2023_485_MOESM1_ESM.docx]

*Additional Material*

Article title

**Dietary patterns (DPs) and metabolic dysfunction-associated fatty liver disease in China’s multiethnic regions**

Xingren zhu^1¶^, Nimaqucuo^2¶^, Ning Zhang^1^, Dan Tang^1^, Yifan Hu^1^, Xiaofen Xie^1^, Qiong Meng^3^, Liling Chen^4^, Xiaoman Jiang^5^, Duojizhuoma^6^, Xing Zhao^1^, Qibing zeng^7*^, Xiong Xiao^1*^

† Joint first authors, these authors contributed equally to this work

*****Joint corresponding authors, Emails: [xiaoxiong.scu@scu.edu.cn. ( X](mailto:xiaoxiong.scu@scu.edu.cn.%20(%20X). X.); [178945324@qq.com](mailto:178945324@qq.com) (Q.Z.)

CONTENTS

[Additional figure 1. Participant flowchart 3](#_Toc100154099)

[Additional Figure 2. The final DAG constructed 4](#_Toc100154100)

[Additional Figure 3. Covariate balance 5](#_Toc100154101)

[Additional figure 4-7. Sensitive analysis 6](#_Toc100154102)

[Additional figure 4. 6](#_Toc100154103)

[Additional Figure 5. 7](#_Toc100154104)

[Additional Figure 6. 8](#_Toc100154105)

[Additional Figure 7. 9](#_Toc100154106)

# Supplementary figure 1. Participant flowchart


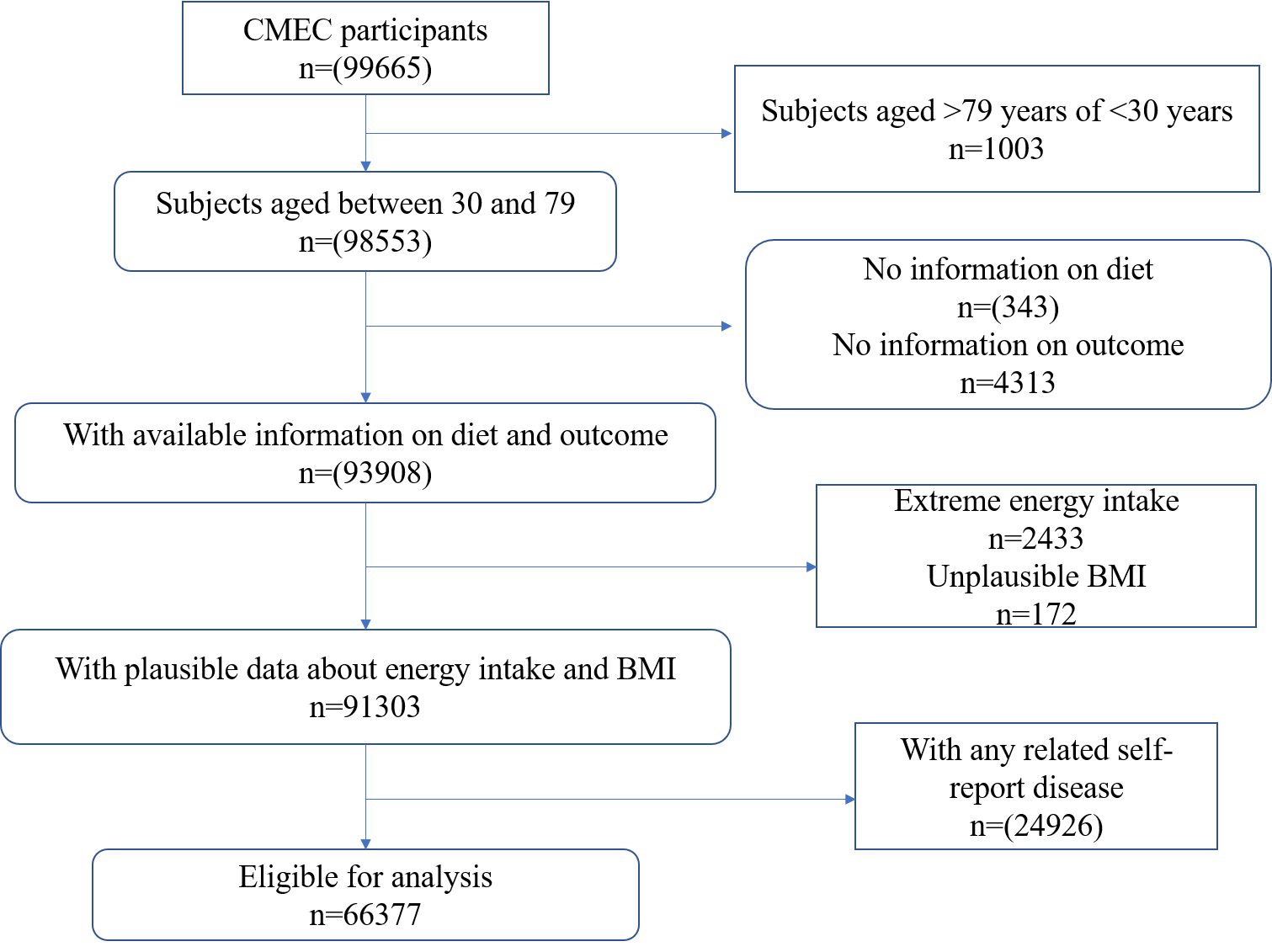


**Supplementary Figure 1. The participants flowchart**

For the current analyses, we focused on participants aged between 30-79 years, so participants out of this range were excluded. We excluded participants whose total energy intake were unplausible (for male: < 800 or > 4200 kcal/day; for female: < 600 or > 3500 kcal/day), as well as those with unplausible body mass index (BMI) (< 14 or > 45 kg/m^2^). And participants with no available information on diet information and outcome information were excluded. Furthermore, to capture a more reasonable casual effect and eliminate the reverse causality, participants with self-reported chronic hepatitis/cirrhosis, coronary heart disease, stroke, hypertension, hyperlipidemia, diabetes, or cancer diagnosed by physician were excluded. Finally, the study sample consists 66377 participants.

# Supplementary Figure 2. The final DAG constructed


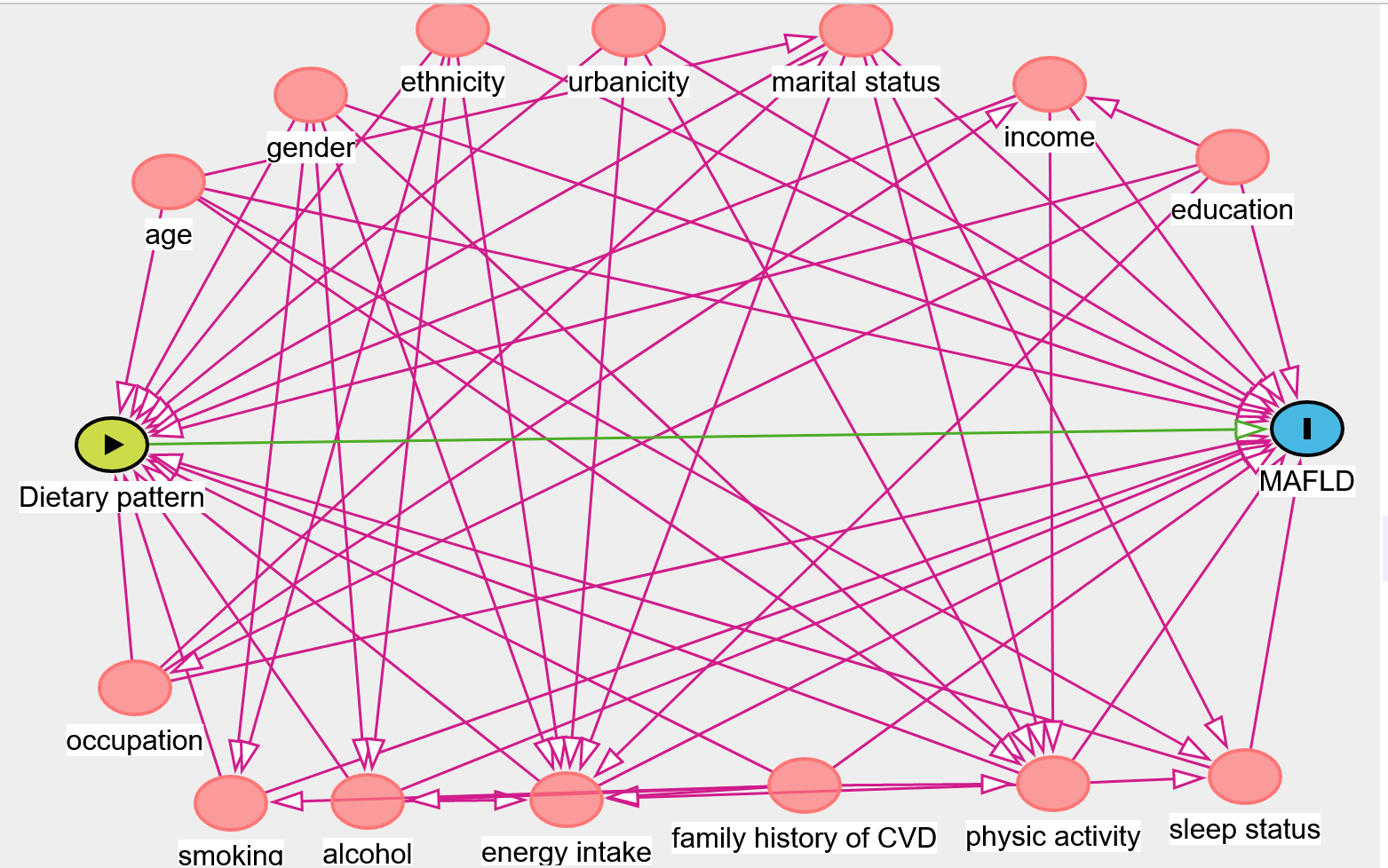


**Supplementary Figure 2. The final DAG constructed**

We constructed DAGs based on the “Evidence Synthesis for Constructing Directed Acyclic Graphs” (ESC-DAGs)[1]. First, we identified all potential confounding between dietary patterns and hypertension/blood pressure based on a systematic literature review. Second, we created a saturated DAG by plotting directed or undirected edges between all variables. Third, we assessed the feasibility of each edge based on three causal criteria (temporality, validity, and theoretical support) to form a simplified DAG. A series of conditional independencies were arising from constructing a DAG. And fourth, we kept performing independence tests and modifying the DAG until all implied conditions in our data were satisfied independently.

DAG was constructed by DAGitty (<http://www.dagitty.net/dags.html>). Finally, sex (male/female), age (continuous), urbanicity (urban/rural), ethnicity (Han/ ethnic minority), marital status (married or not), education (no formal school/primary school/middle and high school/college or university or higher), household income (<¥12000/¥12000-19999/¥20000-5999/¥60000-99999/¥100000-199999/>¥200000), occupation (primary industry practitioner/secondary industry practitioner/tertiary industry practitioner /unemployed), regular smoking (never/former/current), physical activity in metabolic equivalent of task (MET, hours per day), total energy intake (continuous), menopause status for women (premenopausal/perimenopausal/postmenopausal), and family history of CVD(presence/absence), alcohol intake (heavy/light), insomnia symptoms (presence/absence) were adjusted.

Reference

1. Ferguson, K.D.; McCann, M.; Katikireddi, S.V.; Thomson, H.; Green, M.J.; Smith, D.J.; Lewsey, J.D. Evidence synthesis for constructing directed acyclic graphs (ESC-DAGs): a novel and systematic method for building directed acyclic graphs. *Int J Epidemiol* **2020**, *49*, 322-329, doi:10.1093/ije/dyz150.

# Supplementary Figure 3. Covariate balance

**
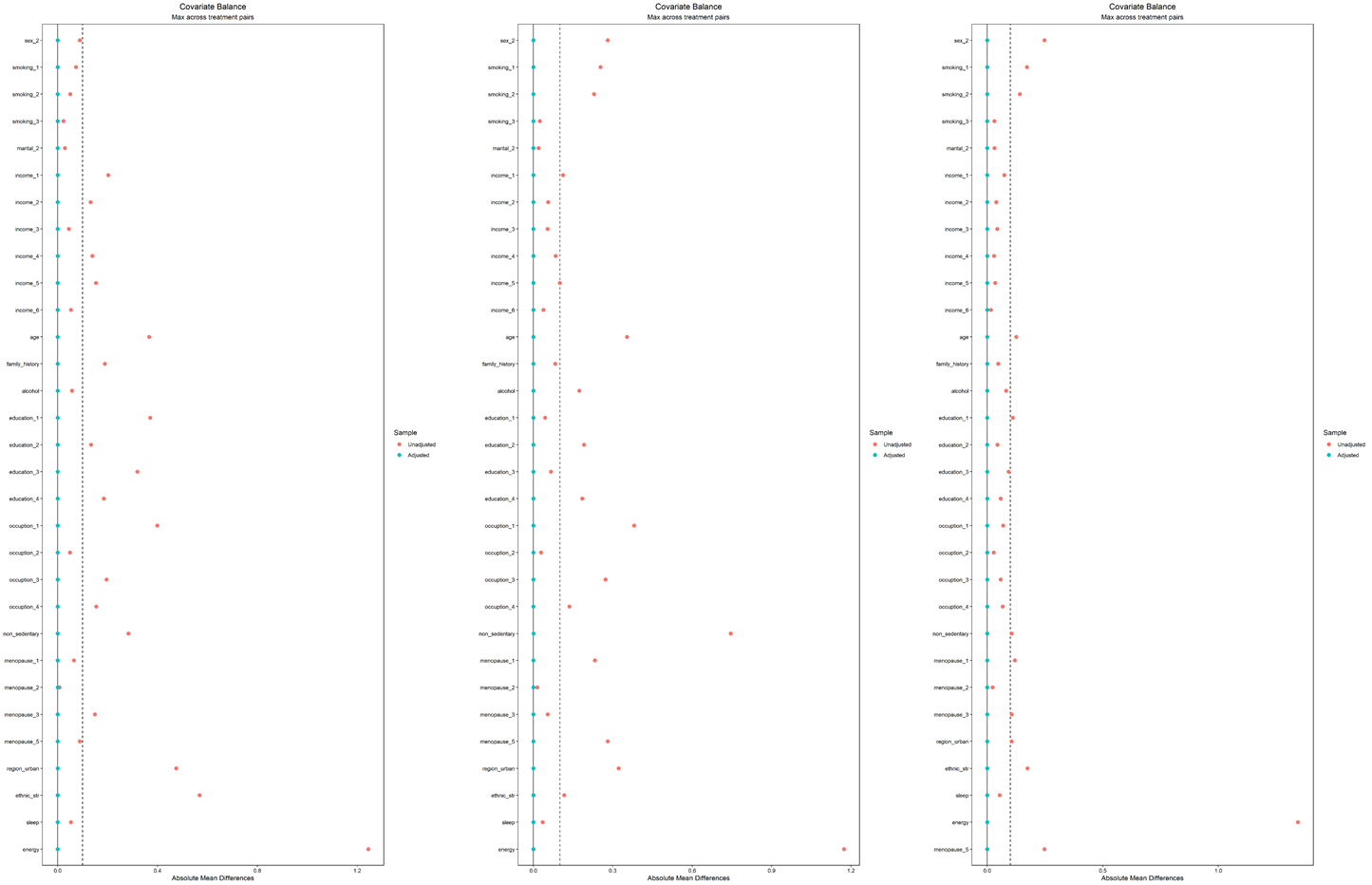
**

**Supplementary Figure 3. Covariate balance**

We conducted covariate balance check based on the R package “cobalt”. As showed above, among all kinds of weighting methods we performed, the entropy balancing showed appropriate balance performance, so we choose entropy balancing as the weighting method in our model.

# Supplementary figure 4-7. Sensitive analysis

Supplementary figure 4. **Estimating the association using traditional logistic regression.**


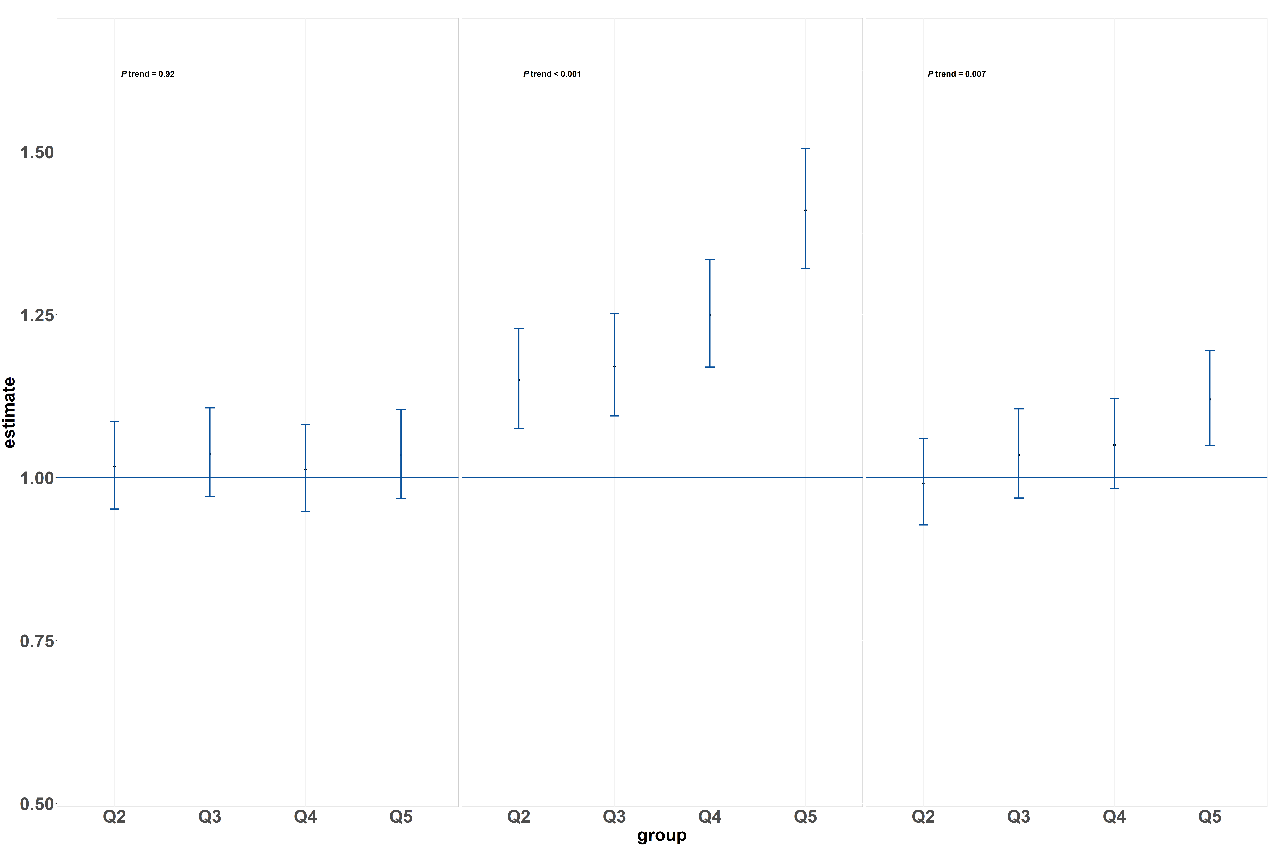


**Supplementary figure 4. Estimating the association using traditional logistic regression.**

In this analysis, we estimated the association using a traditional logistic regression. Overall, the trend is similar and the association is lightly attenuated.

Supplementary Figure 5. Estimating the association with redefined outcome NAFLD.


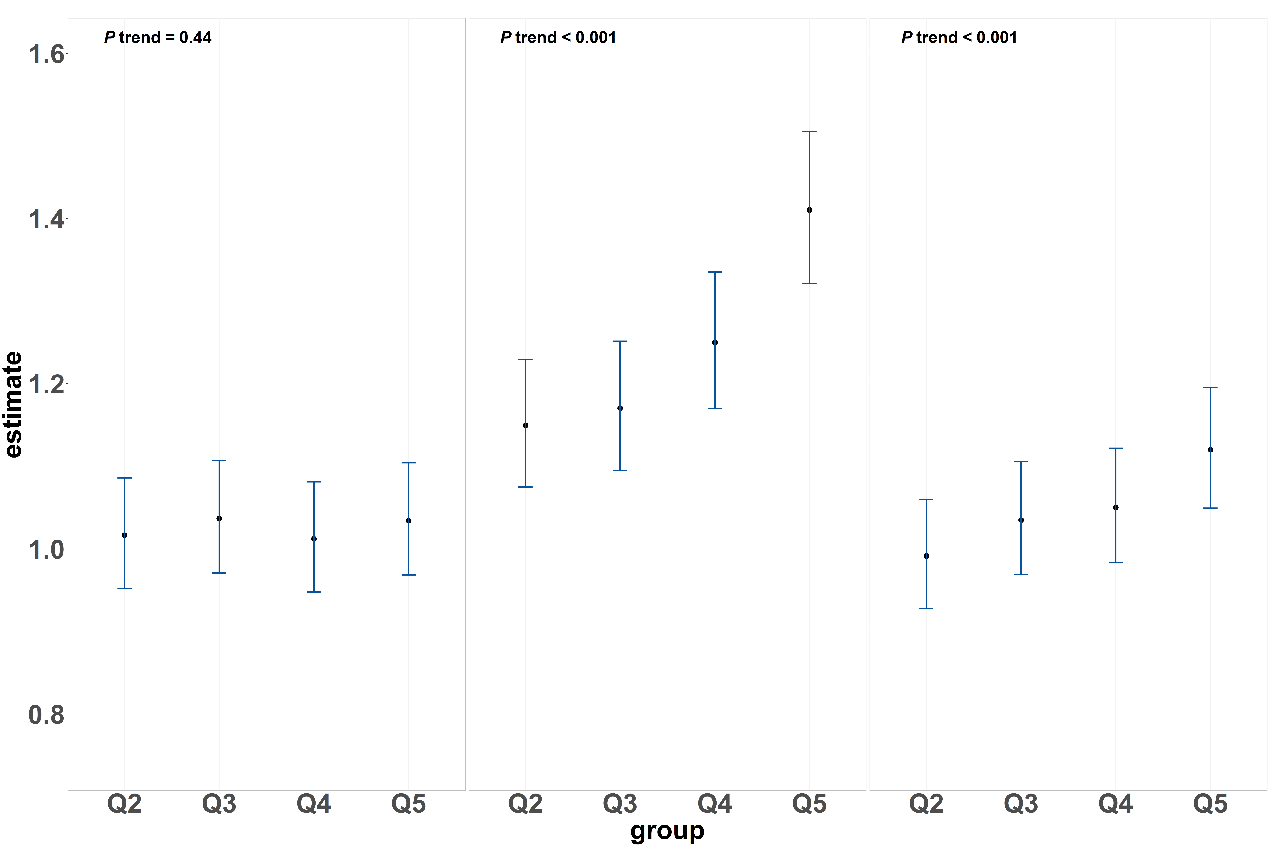


Supplementary Figure 5. Estimating the association with redefined outcome NAFLD.

In this sensitive analysis, we redefined the outcome based on the criteria of NAFLD. As shown above, the result was similar.

Supplementary Figure 6. **Estimating the association without excluding participants with self-reported diseases.**


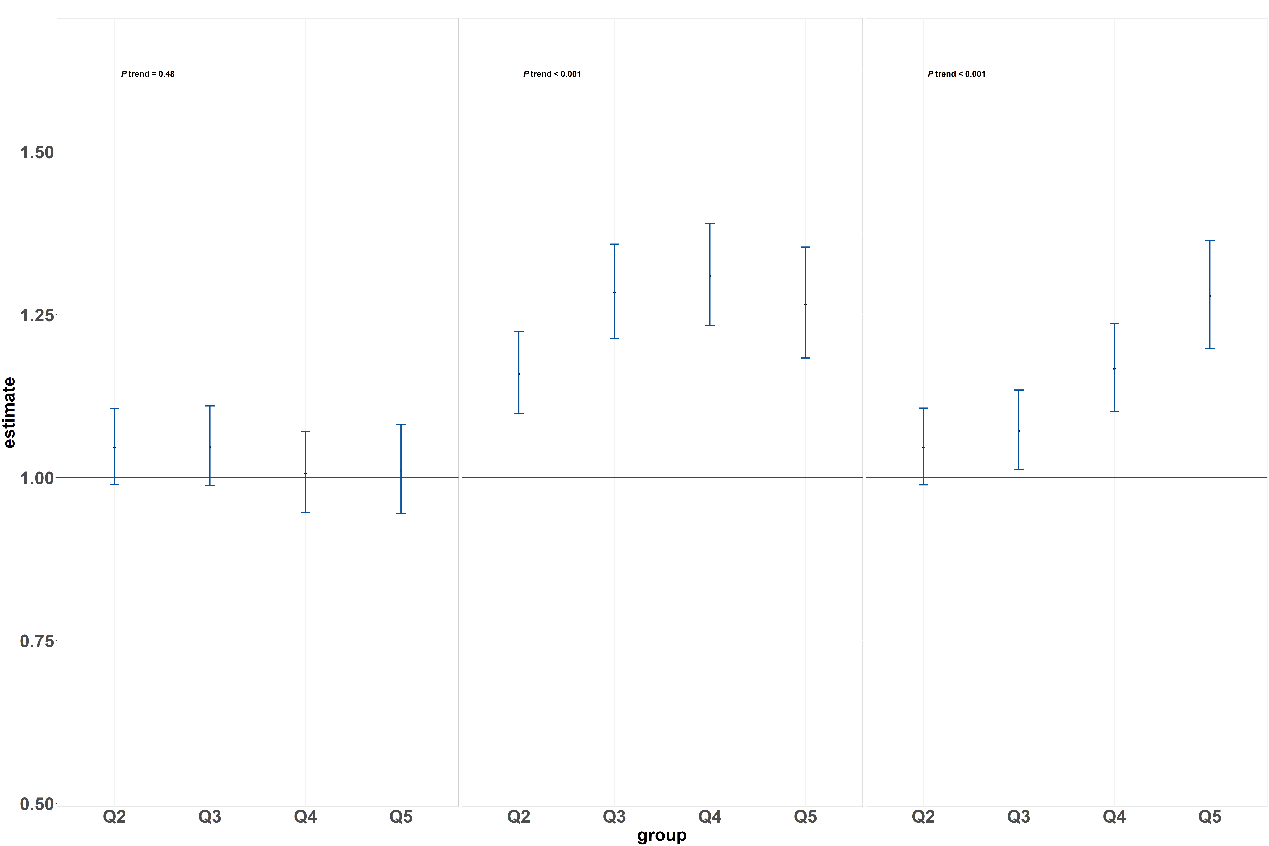


**Supplementary Figure 6. Estimating the association without excluding participants with self-reported diseases.**

In this sensitive analysis, we did not exclude participants with self-reported chronic hepatitis/cirrhosis, coronary heart disease, stroke, hypertension, hyperlipidemia, diabetes, or cancer. As shown above, the association was attenuated but the trend was similar

## Supplementary Figure 7. Estimating the association using complete data.


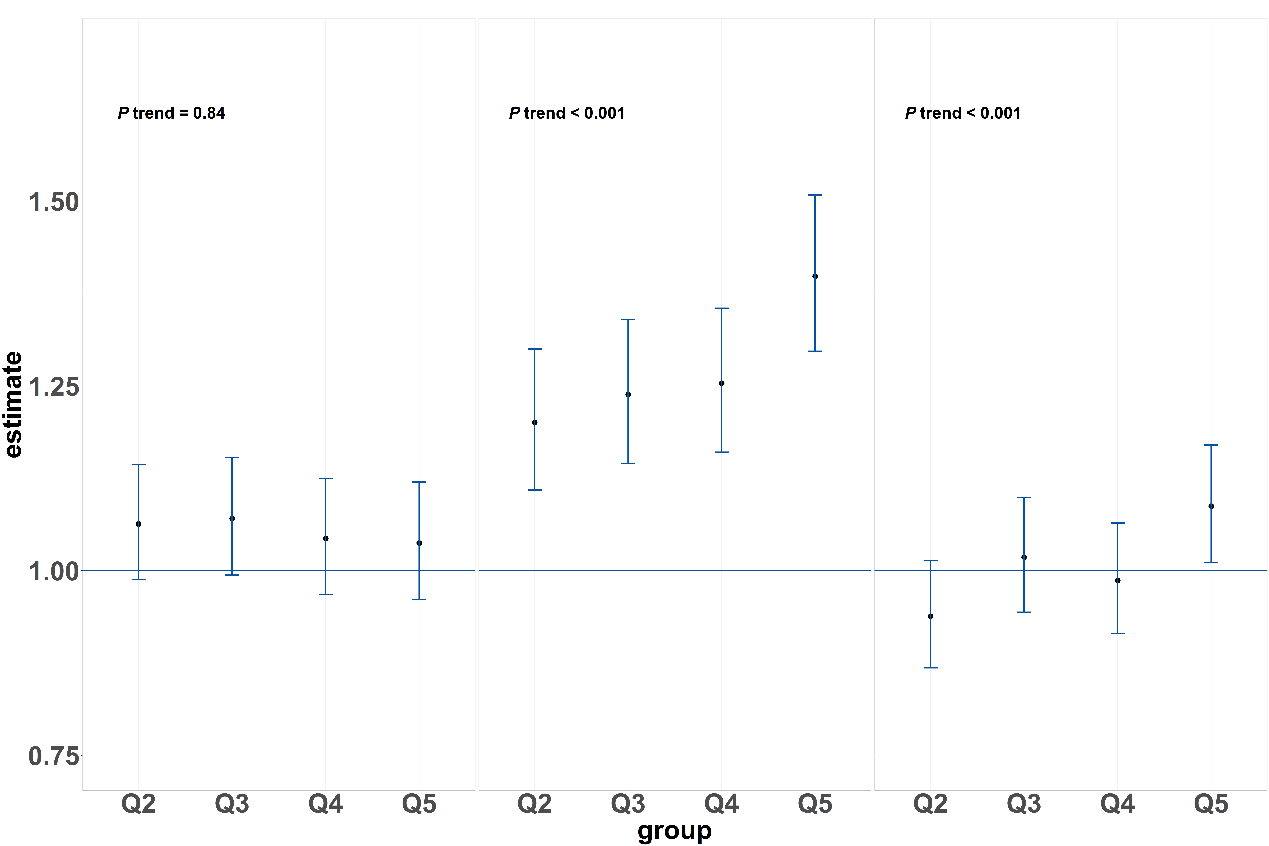
**Supplementary Figure 7. Estimating the association using complete data.**

In this sensitive analysis, we estimated the association using participants with complete information on diet instead of imputation data. As shown above, the association was lightly attenuated.
